# Supplementary material for: Plastic sexual ornaments: Assessing temperature effects on color metrics in a color-changing reptile
Source: PLoS One. 2020 May 20;15(5):e0233221. doi: 10.1371/journal.pone.0233221 (PMC7239470; doi:10.1371/journal.pone.0233221)
Supplement: Attachment — Submitted filename: PLOS One letter to editor_2.docx [file pone.0233221.s002.docx]

**Braulio Assis**	The Pennsylvania State University	Phone - (814) 865-4826

PhD Student	208 Mueller Laboratory	Fax - (814) 865-9131

Department of Biology	University Park, PA 16802	Email – bmd5458@psu.com

March 30, 2020

Dr. Daniel Osorio
Editor

PLOS One

Dear Dr. Osorio,

Thank you for the insightful reviews of our manuscript “Plastic sexual ornaments: assessing temperature effects on color metrics in a color-changing reptile” [PONE-D-19-26164]. We appreciate the time and effort you and the reviewers put into assessing this manuscript. We have made the suggested changes to the manuscript, such as including an analysis of brightness changes (meaning that now we provide a thorough assessment of hue, saturation and brightness changes with temperature for this species) and mean cone excitation values for males and females across treatments. We hope that you now find this manuscript acceptable for publication. Our responses are given below.

Sincerely,

Braulio Assis

-------------------------------------------

**1. I am not aware of any other example of an animal showing such dramatic colour changes with temperature at least in a what is clearly a signalling colour. I guess that you are planning to publish another article on this subject and what to keep your powder dry, but it would would be good to have at least some comment on the generality of the phenomenon, what you see as its significance, and on the literature.**

**Response:** We now acknowledge that dramatic cases of this may in fact be rare, when we discuss other, perhaps relatable, examples from the literature (line 457-467). We also address the potential significance of this trait as signal of thermal performance (lines 449-456).

**2. a) The literature in this area uses the terms: 'purity', 'saturation', and 'chroma' - and maybe even 'chromaticness', more or less vaguely and interchangeably. I think it would be useful to have an explicit definition of the metric used, **preferably supported by a diagram to help those readers not familiar with the details of pavo etc. ****

**Response:** Indeed, several interchangeable terms are found in the literature for the same property of color. To avoid confusion, we now refer to this metric exclusively as “saturation” throughout and elaborate on its meaning (lines 97-106).

**b) For similar reasons it would be nice also to see how the could move in the lizard's receptor space.**

**Response:** We considered including a figure illustrating the hue shifts in a color space. Unfortunately, the data was not as well represented as we hoped given their fine scale of our data in such a wide space. We have included the figure in a preliminary format here, and if you believe it would still be informative to the readers, we would be happy to include it in the manuscript.

**c) No mention is made of 'luminance', yet this may well not be irrelevant to colour appearance, and should I think at least be documented -**

**Response:** We have replicated our analyses of hue and saturation on mean brightness as well and provide a short description of its meaning (lines 340-345). With that, our manuscript now contains information on changes in hue, saturation, and brightness for this species.

**d) Estimated receptor excitation for all colours studied should be documented and available to readers, if this is not already done.**

**Response:** We now provide a table with mean ± SD excitation values for the four cone types, given for males and females and the two temperature treatments (lines 271-274).

**3. Line 67. Structural colours are often not associated with a specialised 'iridophores' but the general chitin, keratin matrix of the epidmermis.**

**Response:** We now address these other possibilities and relevant references (lines 68-69).

**4. Line 98. The relevance/nature of 'allocated pigments' to structural coloration should be made clear, since the pigment if there is one is likely only to be melanin, which is not in short supply for these lizards.**

**Response:** Since color is indeed such a diverse trait with a variety of underlying mechanisms across species, we opted to simply remove this statement altogether.
