# Supplementary material for: Plastic sexual ornaments: Assessing temperature effects on color metrics in a color-changing reptile
Source: PLoS One. 2020 May 20;15(5):e0233221. doi: 10.1371/journal.pone.0233221 (PMC7239470; doi:10.1371/journal.pone.0233221)
Supplement: Attachment — Submitted filename: PLOS One letter to editor_3.docx [file pone.0233221.s003.docx]

**Braulio Assis**	The Pennsylvania State University	Phone - (814) 865-4826

PhD Student	208 Mueller Laboratory	Fax - (814) 865-9131

Department of Biology	University Park, PA 16802	Email – bmd5458@psu.com

April 26, 2020

Dr. Daniel Osorio
Editor

PLOS One

Dear Dr. Osorio,

Thank you for the insightful review of our manuscript “Plastic sexual ornaments: assessing temperature effects on color metrics in a color-changing reptile” [PONE-D-19-26164]. We are grateful for your commitment in helping us improve this manuscript. Your suggestions were pertinent, and we made the recommended additions. Detailed explanations are given below.

Sincerely,

Braulio Assis

-------------------------------------------

**Thank you for submitting your manuscript to PLOS ONE. I am reluctantly asking for a minor revision, because I think that technically this ms does meet the PLoS ONE publication criteria,but there are couple of issues:**

**1. The cover letter states for comment 2b) b)'it would be nice also to see how the could move in the lizard's
receptor space.' that 'the data was not as well represented as we hoped given their fine scale
of our data in such a wide space. We have included the figure in a preliminary format
here....'  I do not see any such preliminary  figure, and I find the justification that the receptor space is too large odd, as it is easy to plot an enlarged fraction of the entire space. Although you argue for the use of  measure of colour (change) independent of the eye I think it is at least useful to know whether the colour changes that you document  a) their detectability; and b) their direction to a natural viewer.**

**Response:** This time we submitted a figure that in our judgment was the most representative of hue and saturation changes in the lizard color space: a two-dimensional projection of the tetrahedral space from the perspective of the UV vertex (figure 3, lines 274-277)

**2. Please comment that   in colour science saturation conventionally refer to an aspect of colour appearance (to human viewers) the usual definition of saturation,  i.e. difference from the most similar grey (Wyszecki & Stiles 1982; or similar elsewhere). The use of the term in the animal coloration literature to refer to some measure of variation in reflectance across the visible spectrum is now established, but somewhat confusing especially where colour appearance to the viewer is relevant.**

**Response:** A statement referencing the definition of saturation in color science as well as our own in animal coloration was included along with the relevant references (Wyszecki & Stiles 1982; Wilkins & Osorio 2019) (lines 97-105; 440).

**3. It seems to me that your model 3, though somewhat arbitrary is at least quite general comapred to measures S1, and perhaps well matched to animal visual systems because rhodopsin spectral sensitivities have a half width of roughly 100nm. Some comment to this effect would be useful.**

**Response:** We mention the biological relevance of this approach due to the Gaussian distribution and sensitivity of cone sensory pigments (lines 410-412).

**4. Are your spectra available as ESM or otherwise?  I think they should be.**

**Response:** We followed this suggestion and decided to upload all the raw spectra (.txt files) along with our dataset and R code in public repositories if this manuscript is accepted for publication.

**As I say these are 'optional' comments and I will accept the ms with any reasonable final revisions.  This is a very interesting system and you have made a thoughtful and careful study.**
